# Preliminary Investigation of Different Drying Systems to Preserve Hydroxytyrosol and Its Derivatives in Olive Oil Filter Cake Pressurized Liquid Extracts

**DOI:** 10.3390/foods10061407

**Published:** 2021-06-18

**Authors:** Lucía López-Salas, Inés Cea, Isabel Borrás-Linares, Tatiana Emanuelli, Paz Robert, Antonio Segura-Carretero, Jesús Lozano-Sánchez

**Affiliations:** 1 Department of Food Science and Nutrition, University of Granada, Campus Universitario S/N, 18071 Granada, Spain; lslucia@correo.ugr.es (L.L.-S.); jesusls@ugr.es (J.L.-S.); 2 Departamento de Ciencia de los Alimentos y Tecnología Química, Facultad de Ciencias Químicas y Farmacéuticas, Universidad de Chile, Casilla 133, Santiago 8380494, Chile; ines.cea@gmail.com (I.C.); proberts@uchile.cl (P.R.); 3 Center for Systems Biotechnology, Fraunhofer Chile Research, Av. Del Cóndor 844 Floor 3, Santiago 8580704, Chile; 4 Functional Food Research and Development Centre (CIDAF), Health Sciencie Technological Park, Avda. Del Conocimiento S/N, 18016 Granada, Spain; ansegura@ugr.es; 5 Department of Food Technology and Science, Center of Rural Sciences, Federal University of Santa Maria, Camobi, Santa Maria 97105-900, RS, Brazil; tatiana.emanuelli@ufsm.br; 6 Department of Analytical Chemistry, Faculty of Sciences, University of Granada, 18071 Granada, Spain

**Keywords:** olive oil byproducts, hydroxytyrosol, PLE, freeze drying, spray drying, HPLC-MS, bioactive compounds

## Abstract

Phenolic compounds present in extra virgin olive oil (EVOO) could be retained in its byproducts during processing. Among them, hydroxytyrosol and its derivatives deserve special attention due to their health benefits recognized by The European Food Safety Authority (EFSA). In the present research, the presence of these compounds in the filter cake byproduct was studied by combining pressurized liquid extraction (PLE) and high-performance liquid chromatography coupled to time-of-flight mass spectrometry (HPLC-TOF-MS). The applied optimum extraction parameters were 1500 psi, 120 °C and aqueous ethanol (50:50, *v*/*v).* The influence of different drying methods (vacuum-, freeze- and spray-drying) in the recovery of phenolic compounds was also evaluated. A total of 16 compounds from EVOO were identified in the extracts, 3 of them being hydroxytyrosol-related compounds, 6 substances of oleoside and elenolic acid derivatives, together with 6 secoiridoids and 1 lignan. The results highlighted the great number of phenolic compounds recovered from filter cake with these techniques, being even higher than the reported content in EVOO and other byproducts. The combination of PLE and freeze-drying resulted in being the best procedure for the recovery of phenolic compounds from filter cake byproduct.

## 1. Introduction

The Mediterranean diet is characterized by high ingestion of non-ultra-processed natural food products such as legumes, fruits, vegetables, cereals, nuts and mainly extra virgin olive oil (EVOO). This dietary pattern has been related to health benefits and protective properties against various pathologies, such as several types of cancer, neurodegenerative and cardiovascular diseases [1,2,3,4,5,6,7,8,9]. Its distinguishing features with respect to other dietary regimes are important from the nutritional and biological point of view: principally, the use of olive oil and the consumption of fruits and vegetables, which are almost absent in many other diets, such as northern Europeans [10].

From all the foods that characterized the Mediterranean diet, EVOO should be highlighted; it is a highly valuable and appreciated product around the world for its beneficial health effects related to its chemical composition. The major compounds in EVOO are triacylglycerols, along with a minor fraction composed of many compounds in very low amounts (around 2% by weight of the oil). These minor substances are mainly tocopherols, pigments, volatile and phenolic compounds [11,12]. In recent years, these phenolic compounds have acquired great importance for their beneficial health properties, as antioxidant, anticancer, estrogenic, antidiabetic, antihypertensive, antithrombotic or anti-inflammatory capacities, among others effects [13,14,15,16,17].

Thus, phenolic compounds naturally present in EVOO have aroused great interest in the scientific world since the European Food Safety Authority (EFSA) recognized their beneficial antioxidant effects for the health at the minimum dose of 5 mg in 20 g of olive oil [18,19,20]. This health potential is associated with its protection against oxidative damage of LDL particles responsible for the transport of lipid molecules [18]. Hydroxytyrosol and its derivatives are considered the most important phenolic compounds present in EVOO due to their strong preventive effects exerted against many pathological processes. In fact, the EFSA authorized the health claim “contribute to the protection of blood lipids from oxidative stress” for EVOO, providing that its content is at least 250 mg/kg of these phenolic compounds.

Most of the Mediterranean countries (especially Spain, Italy and Morocco) produce almost all of the world’s olive oil. In this industrial processing, large quantities of wastes are generated in different steps over the production [1,21]. Industrial processing plants provided with a three-phase decanter generate two types of byproducts, olive-mill wastewater (OMWW or *alpechin*) and pomace (*orujo*, solid waste) [22,23]. On the other hand, the two-phase decanter plants generate *alperujo* as the main byproduct, which is made up of a combination of solid and liquid wastes (*orujo* and *alpechin*)) [4,23]. Nevertheless, other byproducts generated during EVOO production are liquid wastes and cakes, which are solid and liquid wastes from EVOO storage and filtering, apart from twigs and leaves [12,23]. Additionally, in the EVOO industrial processing, a partition of phenolic compounds present in the fruit between the EVOO and the generated byproducts occurs, leading to a transfer of these compounds from the EVOO to the olive oil wastes.

In this sense, filter cakes are generated in large amounts in the final step of cloudy oil filtration. The main objective of the filtration process is to remove humidity and suspended solids of EVOO in order to obtain good quality oil for consumer acceptance, as well as to improve oil stability [24]. The loss of water leads to a decrease in polar compounds content in EVOO, including phenolic compounds. This fact originates filter cakes enriched in these compounds of interest with much potentials, such as hydroxytyrosol and derivatives [11]. Therefore, these byproducts could be used as a source of bioactive compounds for valued applications, reducing the economic and environmental impact of the waste treatment in the industry, enabling a more sustainable EVOO production inside a circular economy approach.

In this line, the main objective of this research was to explore the potential combination of an advanced extraction technique (pressurized liquid extraction, PLE) coupled to vacuum-, freeze- and spray-drying systems to obtain the best innovative process to recover hydroxytyrosol and its antioxidant derivatives from filter cake generated during EVOO production. The above-mentioned techniques were complemented by a deep chemical characterization of the extracted bioactive compounds carried out by high-performance liquid chromatography coupled with time-of-flight mass spectrometry (HPLC-ESI-TOF-MS). According to our knowledge, this is the first time that a detailed quantitative characterization of individual phenolic compounds recovered from a filter cake by the combination of PLE with different drying alternatives was evaluated.

## 2. Materials and Methods

### 2.1. Reagents

The chemicals employed during the development of this research were of analytical grade reagent. Milli-Q water for PLE and mobile phase preparation was obtained with a Millipore system (Bedford, MA, USA). Ethanol was provided by VWR Chemicals (Radnor, PA, USA), while Fisher Chemicals (Waltham, MA, USA) supplied cellulose filters and sand for PLE experiments. The mobile phase composition was obtained with acetic acid supplied by Sigma–Aldrich (Steinheim, Germany) and LC-MS-grade acetonitrile from Fisher Chemicals (Waltham, MA, USA). The commercial standards for quantitation purposes (quinic acid, hydroxytyrosol, pinoresinol, and oleuropein) were acquired from Sigma–Aldrich (St. Louis, MO, USA), Arbo Nova (Turku, Finland) and Extrasynthese (Lyon, France).

### 2.2. Olive Oil Filter Cake Samples

Olive fruits (*Olea europea* L.) from the Arbequina variety were collected in Granada (Andalucía, Spain) in December 2020. The olives were processed without storage by continuous industrial plants equipped with a hammer crusher, a horizontal malaxator, and a two-phase decanter (Aceites Maeva S.L. production plant). The EVOO (25.000 L) was filtered at a constant flow (5.000 L/h) and an ambient temperature with industrial filtration equipment. This machinery is composed of a filter tank and a filter aid composed of 50 kg of cellulose fiber (Spindacel, AEB Ibérica, Barcelona, Spain). The filter cake samples were obtained after the EVOO filter process. In order to obtain representative results without the influence of several factors in the phenolic content, the phenolic extract was isolated from this matrix immediately after filtration.

### 2.3. Pressurized Liquid Extraction

Filter cake PLE extracts were obtained by an accelerated solvent extractor (ASE 350, Dionex, Sunnyvale, CA, USA). Phenolic compounds were recovered from the filter cake sample (8 g), performing the extraction with optimum parameters. The extraction conditions were: 1500 psi, 120 °C and aqueous ethanol (50:50, *v/v*). These conditions were previously optimized for olive oil filter cakes in previous work, in which different combinations of ethanol percentages (0–85%, ethanol:water, *v/v*) and temperatures (40 to 175 °C) were tested [12].

Before the PLE experiments, water and ethanol were submitted to sonication for 15 min to remove the dissolved oxygen. Each extraction was performed inside 33 mL extraction cells containing 8 g of filter cake mixed homogeneously with 2.5 g of sand. Before polar compounds extractions, a clean-up step was carried out using n-hexane (1500 psi without heating) to remove the lipophilic fraction from the filter cake. The PLE experiment starts with heating the extraction cell until the pre-fixed extraction temperature. After that, the recovery of phenolic compounds was performed during a static extraction time of 20 min. The procedure was carried out in triplicate in order to assure reproducibility. The obtained extracts were protected from light and maintained at −20 °C until further processing. For solvent removal, three different drying systems were applied following the procedures described in the next section.

### 2.4. Vacuum-, Freeze- and Spray-Drying

Vacuum-drying of hydro-alcoholic filter cake extract. The PLE extracts were dried under a vacuum in a Savan SC250EXP Speed-Vac (Thermo Scientific, Leicestershire, UK) operating at 13 kPa and 35 °C.

Spray-drying of hydro-alcoholic filter cake extract. A laboratory spray-dryer (4M8-TriX Spray-Dryer, Procept, Zelzate Belgium) equipped with a peristaltic pump; a bifluid nozzle; a heater; drying, atomizing and cyclone gases (air); and a dry product collector was used for the spray-drying process. The drying parameters were: inlet air flow (0.35 m^3^/min), air flow atomizing nozzle (0.013 m^3^/min), inlet and outlet air temperatures (110 and 49 °C, respectively), pump speed (2 mL/min) and differential pressure over cyclone (15 mBar).

Freeze-drying of hydro-alcoholic filter cake extract. The freeze-drying process was carried out in a laboratory-scale freeze-dryer (Virtis SP Scientific, Thermo Fisher, Spain). Primary drying began with P = 400 Torr and T = −45 °C and ended at atmospheric pressure (AP) and T = 0 °C. Secondary drying had a starting point with AP and T = 0 °C, and a set point with AP and T = 25 °C. Each drying system was carried out in triplicate.

### 2.5. HPLC-ESI-TOF-MS Analysis

The analysis of PLE extracts phenolic compounds from filter cake samples obtained by vacuum-, freeze- and spray-drying processes were performed with a high-performance liquid chromatographer combined with mass spectrometry using a time-of-flight mass analyzer (HPLC-ESI-TOF-MS). The samples were reconstituted in the extraction solvent, aqueous ethanol mixture (50%), at a concentration of 10 mg/mL, and filtered through a 0.2 µm syringe filter of regenerated cellulose. For quantitation purposes, working solutions of commercial standards were prepared at concentrations of 1 mg/mL in the same solvent used for filter cake extracts. Calibration curves with six concentration levels (*n* = 6) were prepared in triplicate by serial dilution of the working solutions until concentrations of 0.5; 1.5; 5; 10; 15 and 20 μg/mL. The calibration levels and samples were injected in triplicate, and the compound concentrations were determined using the area of each individual compound (three replicates) and by interpolation in the corresponding calibration curve.

The HPLC analysis was carried out in an RRLC 1200 system (Agilent Technologies, Palo Alto, CA, USA), equipped with a vacuum degasser, a binary pump, an automated sampler and a thermostatically controlled column compartment according to a previously described analytical method [25]. The samples were separated on a Zorbax Eclipse Plus C18 analytical column (1.8 µm, 4.6 × 150 mm) from Agilent Technologies (Palo Alto, CA, USA). The mobile phases consisted of water with 0.25% acetic acid (phase A) and methanol (phase B) using a gradient elution according to the following profile: 0 min, 5% B; 7 min, 35% B; 12 min, 45% B; 17 min, 50% B; 22 min, 60% B; 25 min, 95% B, 27 min, 5% B. The initial conditions were maintained for 5 min before the next injection. The flow rate was 0.5 mL/min, the column temperature, 25 °C, and the injection volume, 10 µL.

Detection was performed using a microTOF II mass spectrometer from Bruker Daltonik within a mass range of 50–1000 *m*/*z* operating in negative ion mode. The instrument was equipped with an ESI interface from Agilent Technologies. Nitrogen was used as drying and nebulizing gas. The operating parameters were as follows: drying gas flow rate, 9 L/min; drying gas temperature, 190 °C; nebulizer, 2 bar; capillary, 4000 V; End Plate Offset, −500 V; Capillary exit voltage, −120 V; Skimmer 1, −40 V; Hexapole 1, −23 V; Hexapole RF, 50 Vpp; Skimmer 2, −22.5 V; Lens 1 transfer, 50 µs and Lens 1 Pre-Pulse Storage, 3 µs.

In order to recalibrate the mass spectra acquired during the analysis to achieve accurate mass measurements with a precision of 5 ppm, a 5 mM sodium acetate solution was used as a calibrant in the quadratic þlus high-precision calibration (HPC) regression model. All data acquisition and processing operations were controlled with HyStar 3.2 and Data Analysis 4.0 software, respectively (Bruker Daltonics, Bremen, Germany).

### 2.6. Statistical Analysis

Data were statistically processed using Origin (Version Origin Pro 8.5, Northampton, MA, USA). For this data set, a one-way analysis of variance (ANOVA, Tukey’s test) at a 95% confidence level (*p* ≤ 0.05) was performed to point out the differences in quantitative bioactive compounds contents found between PLE EVOO filter cake samples with statistical significance.

## 3. Results

### 3.1. Identification of Polar Compounds in Olive Oil Filter Cake PLE Extracts by HPLC-ESI-QTOF-MS

**Figure 1:**
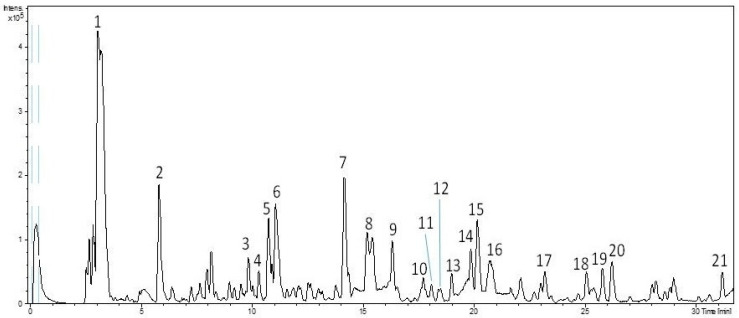
Base–peak chromatograms (BPC) of the freeze-drying hydro-alcoholic filter cake extract obtained by high-performance liquid chromatography coupled with time-of-flight mass spectrometry (HPLC-ESI-TOF/MS).

Targeted compounds were identified by the interpretation of their MS spectra and the molecular formula provided by the software, together with data previously reported in the literature. The base–peak chromatograms (BPC) of the freeze-drying PLE filter cake extract obtained by HPL-ESI-TOF-MS acquired in negative polarity is presented in Figure 1. The tentatively identified phenolic compounds are summarized in Table 1, including retention time, experimental and calculated *m*/*z*, molecular formula, error (ppm), and the samples in which the compounds were previously described.

The identification was performed by a comparison of the retention time and mass spectra provided by the analytical platform with the data of commercial standards when available. The rest of the compounds with an unobtainable standard were identified by the interpretation of their mass spectra provided by the TOF–MS instrument and the information previously reported in the bibliography.

A total of 21 compounds were identified in the hydro-alcoholic PLE extracts, 4 of them remaining unknown despite the efforts made for their identification (UK 1–4). Among these, three substances belong to phenolic alcohols family or derivatives, whereas six compounds were secoiridoids and only one lignan. The rest of the putative compounds could not be considered phenolic structures. Concretely, six compounds were characterized as oleoside and elenolic acid derivatives and the last one was identified as quinic acid.

All the identified compounds were found in the entire collection of PLE extracts evaporated under vacuum, spray-drying and freeze-drying. Peak 1, with *m*/*z* 191 and molecular formula C_7_H_12_O_6_, was identified as quinic acid [26]. With regard to phenolic alcohols or derivatives, peak 2 and peak 4 were identified as oxidized hydroxytyrosol and hydroxytyrosol, respectively. The first one was previously described in olive oil and different byproducts: (a) filter cake produced over the filtration process of Picual EVOO, (b) olive oil pomace and (c) solid and aqueous wastes generated during the industrial storage of Hojiblanca EVOO variety [12,27,28]. In these studies, only a vacuum system was applied as the post-extraction drying procedure. On the other hand, it is commonly known that hydroxytyrosol was widely described in different olive oils and its byproducts [11,29,30,31]. Finally, peak 15, with a retention time of 20.13 min, was identified as hydroxytyrosol acetate, a compound previously characterized in olive oil samples [30].

Furthermore, six compounds were identified as secoiridoids and their derivatives. In detail, peak 6, eluting at a retention time of 11.07 min and displaying an *m*/*z* of 407, was characterized as a secoiridoid derivative. This compound was previously reported in olive pomace [27]. Peak 9, with *m*/*z* 377 and 16.30 min, was identified as oleuropein aglycone derivative according to the literature [28,32]. Peak 16, with *m*/*z* 393 and molecular formula C_19_H_22_O_9_, was proposed as hydroxy oleuropein aglycon, and peak 19, at a retention time of 25.75 min, was identified as hydroxy decarboxymethyl-ligstroside aglycone [28,33]. Moreover, peaks 20 and 22 were also characterized as compounds belonging to the secoiridoid group. The first one, with a retention time of 26.17 min and *m*/*z* 535, was determined to be comselogoside (p-coumaroyl-6-secologanoside), which was identified in olive oil and its byproducts (alperujo and olive mill wastewater) [4,18,31]. Peak 22, with *m*/*z* 557, was characterized as 6-O-[(2E)-2,6-dimethyl-8-hydroxy-2-octenoyloxy] secologanoside. This compound has been described in olive leaf extracts by other authors [34]. Finally, peak 18, with *m*/*z* 415, was identified as (+)-acetoxypinoresinol, a lignan [4,28].

**Figure 2:**
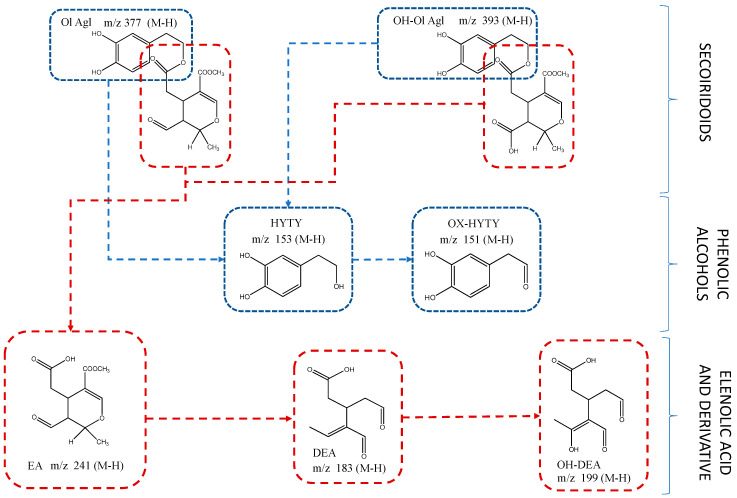
Chemical structures of the main phenolic compounds and their derivatives: Ol Agl, Oleuropein aglycone; OH-Ol Agl, Hydroxy oleuropein aglycone; HYTY, hydroxytyrosol; OX-HYTY, Oxidized hydroxytyrosol; EA, elenolic acid; DEA, Dialdehydic form of decarboxymethyl-elenolic; OH-DEA, Hydroxylated product of the dialdehydic form of decarboxymethyl-elenolic acid.

With regard to non-phenolic molecules, peak 7, with *m*/*z* 389 and molecular formula C_16_H_22_O_11_, corresponded to oleoside/secologanoside. These substances were previously found in olives and olive pomace [27,32,35]. Other related compounds characterized in these PLE extracts consist of elenolic acid derivatives. Thus, peak 3 was proposed as the hydroxylated product of the dialdehydic form of decarboxymethyl-elenolic acid; and peak 11, displaying an *m*/*z* 215 and retention time of 18.06 min, was characterized as the aldehydic form of decarboxymethyl elenolic acid [28]. Peak 5, at a retention time of 10.9 min and *m*/*z* 183, was determined to be the dialdehydic form of decarboxymethyl-elenolic acid [33]. Peaks 10 and 14, both with *m*/*z* 241 and the same molecular formula C_11_H_14_O_6_, were identified as elenolic acid or its isomer [29]. In addition, Figure 2 includes the chemical structure of the main phenolic alcohols, secoiridoids and their derivatives identified in the studied extracts.

### 3.2. Effect of Freeze-, Vacuum- and Spray Drying on the Content of Hydroxytyrosol, Its Derivatives and Other Phenolic Compounds

**Figure 3:**
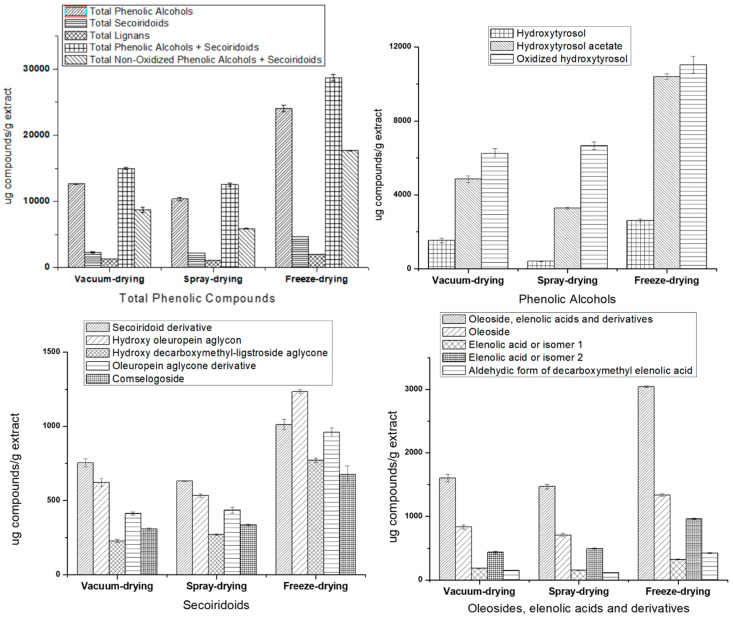
Concentrations of phenolic compounds characterized by HPLC-ESI-QTOF-MS in filter cake PLE extracts with different drying systems.

The quantification of phenolic and other polar compounds identified in the EVOO filter cake extracts was carried out using the four commercial standards described above. All the calibration curves showed good linearity, better than 0.98 (Table S1). Therefore, hydroxytyrosol and quinic acid were quantified by the calibration curves obtained with their respective commercial standards. The other phenolic compounds, for which no commercial standard was available, were tentatively quantified using standards with a similar structure. Oxidized hydroxytyrosol and hydroxytyrosol acetate were quantitated with the hydroxytyrosol calibration curve. Secoiridoids and their derivatives (secoiridoid derivative, hydroxy oleuropein aglycon, oleuropein aglycone derivative, hydroxy decarboxymethyl-ligstroside aglycone and comselogoside) were quantified using the oleuropein calibration curve. Oleosides, elenolic acid and derivatives were also quantified using the oleuropein calibration curve. Finally, (+)-acetoxypinoresinol (a lignan) was quantified using the pinoresinol standard (Table S2). Figure 3 shows the total content of each family as well as the individual concentration of the compounds belonging to different families: phenolic alcohols, secoiridoids, and oleoside/elenolic acid and their derivatives.

Concerning total phenolic compounds content (TPC), as expected, the concentrations of these compounds in freeze-dried PLE extract were higher compared to the other drying PLE extracts. In addition, freeze-dried PLE extract also had the highest amount of acetoxypinoresinol, the unique lignan identified in the studied extracts. The second drying system that provides a higher TPC value for the PLE extract was vacuum-drying. Finally, the drying system with the lowest amount of total phenolic compounds was spray-drying.

From the point of view of individual contents of phenolic alcohols, secoiridoids and their derivatives, freeze-drying reported the best concentrations for all these compounds. Moreover, regarding hydroxytyrosol and its derivatives, the spray-drying system reported the lowest value of hydroxytyrosol of this sample batch. In addition, the oxidized hydroxytyrosol/hydroxytyrosol ratio in this extract was 3.9- and 3.7-times higher than those obtained for vacuum- and freeze-dried PLE extracts, respectively. Consequently, it could be assumed that the application of spray-drying increases the oxidation of hydroxytyrosol, increasing the concentration of the oxidized product. In fact, it is well-known that these phytochemicals may undergo modifications, and the appearance of phenolic oxidated products could indicate their degradation during the drying process. In spite of the other drying techniques that allowed the preservation of hydroxytyrosol, oxidized hydroxytyrosol was also quantitated in high amounts in both vacuum- and freeze-dried PLE extracts. The latest also showed the greatest amount of hydroxytyrosol acetate. Despite the drying procedure affects the final concentration of oxidized derivatives, it is important to remark that other factors could contribute to the final amount of these compounds in the extracts. In fact, the monitorization of the oxidized hydroxytyrosol in EVOO has pointed out the presence of this compound without EVOO storage as well as an increase in its concentration over the shelf life [28].

Concerning secoiridoids and their derivatives concentrations, the combination of PLE and freeze-drying was also the best procedure to obtain a secoiridoids-enriched extract. Both chemical groups, phenolic alcohols and secoiridoids, were recognized by an EFSA health claim. Table S2 summarizes the relationship among phenolic alcohols, secoiridoids and their oxidized derivatives. It must be taken into account that the identified secoiridoids (except hydroxy decarboxymethyl-ligstroside aglycone) are composed of hydroxytyrosol as phenolic moiety (see Figure 2). The total amount of non-oxidized phenolic alcohols (hydroxytyrosol plus hydroxytyrosol acetate) in freeze-drying PLE extract was 2 and 3.5 times higher than in vacuum- and spray-drying PLE extracts, respectively. Similar results were also observed for total phenolic alcohols and secoiridoids and total non-oxidized phenolic alcohols, plus secoiridoids in all the drying techniques applied in this study. The statistical treatment reported significant differences among the concentrations obtained for the different drying systems (Table S3).

With respect to other chemical compounds identifies in the samples, oleoside was obtained in greater amount in the freeze-drying PLE extract. The family of elenolic acid and derivatives showed little variation in their contents between the three drying systems, but in most cases, they were statistically significant. In all dried extracts, isomer 2 was quantified in higher quantity compared to isomer 1, so the drying technique did not seem to influence this aspect.

Regarding the present study, the quantified amounts of total phenolic alcohols varied from 10,364 to 24,066 mg/kg of the extract, depending on the drying technique, whereas the content of total secoiridoids was from 2202 to 4654 mg/kg of dried extract. Specifically, hydroxytyrosol presented a concentration range from 421 to 2620 mg/kg extract, oxidized hydroxytyrosol from 6258 to 11,041 mg/kg of extract and hydroxytyrosol acetate from 3284 to 10,405 mg/kg extract. The content of hydroxytyrosol and derivatives recovered from the filter cake byproduct in this study is much higher than the values obtained for olive oil samples and other byproducts by several authors [25,26,27,36,37]. One of the studies [25] analyzed the phenolic content of different varieties of EVOO (cv. Hojiblanca, Picual, Cornezuelo, Manzanilla and Arbequina). The phenolic alcohol contents obtained in this research were: (a) from 11 to 12 mg/kg Hojiblanca olive oil; (b) from 9 to 17 mg/kg Picual olive oil; (c) 5 mg/kg Cornezuelo olive oil; (d) 15 mg/kg Manzanilla olive oil and (e) from 5 to 8 mg/kg Arbequina olive oil. In relation to hydroxytyrosol and hydroxytyrosol acetate concentrations, the values were: (a) 6 mg hydroxytyrosol/kg and 0.7 mg hydroxytyrosol acetate/kg Hojiblanca olive oil; (b) from 5 to 11 mg hydroxytyrosol/kg and from 0.6 to 0.7 mg hydroxytyrosol acetate/kg Picual olive oil; (c) 1 mg hydroxytyrosol/kg and 0.6 mg hydroxytyrosol acetate/kg Cornezuelo olive oil; (d) 10 mg hydroxytyrosol/kg and 0.8 mg hydroxytyrosol acetate/kg Manzanilla olive oil; and finally, (e) from 2 to 4 mg hydroxytyrosol/kg and from 1 to 3 mg hydroxytyrosol acetate/kg Arbequina olive oil. Moreover, the secoiridoid amounts were: (a) from 286 to 379 mg/kg Hojiblanca olive oil; (b) from 341 to 570 mg/kg Picual olive oil; (c) 286 mg/kg Cornezuelo olive oil; (d) 417 mg/kg Manzanilla olive oil; and (e) from 113 to 269 mg/kg Arbequina olive oil. On the other hand, another study reported total phenolic alcohols and hydroxytyrosol contents in the range of 6 to 11 mg/kg and 4 to 7 mg/kg of EVOO in the Oueslati variety, respectively [26]. In this study, the secoiridoids content was ranged from 1200 to 2886 mg/kg. Finally, Andjelkovic and collaborators analyzed the phenolic content of different samples of Aglandau, Tanche, Picual, Verdial and Cornicabra EVOO varieties [36]. With respect to Aglandau olive oil, phenolic alcohols were quantified in 17 to 49 mg/kg, hydroxytyrosol concentration from 2 to 24 mg/kg, and secoiridoids content from 42 to 64 mg/kg. Concerning Tanche olive oil, phenolic alcohols were estimated in a concentration range from 23 to 81 mg/kg, hydroxytyrosol from 11 to 49 mg/kg and secoiridoids content from 51 to 58 mg/kg. In Cornicabra olive oil, phenolic alcohols were quantified in 2 to 71 mg/kg, hydroxytyrosol from 2 to 30 mg/kg and secoiridoids content was from 21 to 59 mg/kg. With regard to Picual olive oil, phenolic alcohols presented a concentration from 13 to 54 mg/kg, hydroxytyrosol from 4 to 38 mg/kg and secoiridoids from 31 to 92 mg/kg. Lastly, in Verdial olive oil, phenolic alcohol content varied from 33 to 78 mg/kg, hydroxytyrosol from 12 to 35 mg/kg and secoiridoids from 7 to 72 mg/kg. In all these oil varieties, it can be observed that the phenolic alcohols and secoiridoids contents are much lower than the amounts reported in the present study for filter cake PLE extracts. According to the EFSA recommendations, PLE filter cake samples would be an interesting source of these compounds.

Regarding other byproducts, the phenolic alcohol hydroxytyrosol and oxidized hydroxytyrosol were also identified and quantified in olive pomace extracts obtained under different PLE extraction conditions [27]. This study reported amounts for these compounds in the range from 0 to 675.6 mg/kg of olive pomace. Specifically, the oxidized hydroxytyrosol presented a concentration from 10 to 458 mg/kg extract and the hydroxytyrosol from 22 to 258 mg/kg extract. This means from 626- to 24-times less oxidized hydroxytyrosol and from 19- to 10-times less hydroxytyrosol than the different dried filter cake extracts studied in the present research. Secoiridoids were also recovered in a range from 103 to 517 mg/kg olive pomace extract, indicating a concentration from 21- to 9-times lower than the one reported for filter cake extracts. Finally, another research evaluated the phenolic compounds profile from olive pomace byproducts generated by the treatment of La Pepa and Severini olive varieties [37]. In pomace from La Pepa olive oil, the content of phenolic alcohols was 31 mg/kg, 10 mg/kg of hydroxytyrosol and 130 mg/kg of secoiridoids. Finally, concerning Severini pomace, 30 mg/kg of phenolic alcohols, 8 mg/kg of hydroxytyrosol and 131 mg/kg of secoiridoids were quantified.

## 4. Conclusions

The recovery of EVOO phenolic compounds from filter cake provides a potential source of bioactive substances, mainly hydroxytyrosol and its derivatives, with a recognized EFSA health claim. However, it should be taken into account that the chosen olive oil variety could affect the phenolic content of this byproduct as it is related to EVOO composition. This study has reported the potential application of PLE coupled to vacuum-, freeze- and spray-drying to obtain phenolic- and secoiridoids-enriched extracts. A marked influence of the drying process on the composition of these extracts was noticed. Therefore, the PLE extracts submitted to freeze-drying showed a higher amount of phenolic compounds compared to other drying techniques. Nevertheless, these preliminary results obtained for all the studied drying systems have pointed out a higher recovery of phytochemicals in the PLE-dried filter cake extracts in comparison with olive oil and other olive oil byproducts considering the information reported in the literature. Future trends directed to develop an efficient scale-up of this procedure could provide a useful tool for the nutraceutical industrial producers, according to the EFSA health claim.

## Figures and Tables

**Table 1:** Phenolic and other polar compounds of extra virgin olive oil (EVOO) filter cake pressurized liquid extraction (PLE) extracts characterized by HPLC-ESI-TOF-MS.

Peak	RT (min)	Proposed Compound	*m*/*z*	*m/z Exp*	Molecular Formula	Error (ppm)	Matrix in Which the Compound Was Previously Described
**1**	3.06	Quinic acid	191.0561	191.0565	C_7_H_12_O_6_	−1.9	Olive oil
**2**	5.82	Oxidized hydroxytyrosol	151.0401	151.0400	C_8_H_8_O_3_	−0.6	Olive oil and olive oil byproducts
**3**	9.85	Hydroxylated product of the dialdehydic form of decarboxymethyl-elenolic acid	199.0612	199.0618	C_9_H_12_O_5_	−0.3	Olive oil and olive oil byproducts
**4**	10.32	Hydroxytyrosol	153.0557	153.0558	C_8_H_10_O_3_	−0.4	Olive oil and olive oil byproducts
**5**	10.9	Dialdehydic form of decarboxymethyl-elenolic acid	183.0663	183.0660	C_9_H_12_O_4_	1.3	Olive oil
**6**	11.07	Secoiridoid derivative	407.1559	407.1568	C_17_H_28_O_11_	−2.3	Olive oil by-products
**7**	14.16	Oleoside/secologanoside	389.1089	389.1099	C_16_H_22_O_11_	2.4	Olives and olive oil byproducts
**8**	15.16	UK 1	409.1140	409.1143	C_19_H_22_O_10_	−0.7	-
**9**	16.30	Oleuropein aglycone derivative	377.1453	377.1456	C_16_H_26_O_10_	−0.7	Olive oil, olives and byproducts
**10**	17.69	Elenolic acid or isomer 1	241.0718	241.0722	C_11_H_14_O_6_	−1.8	Olive oil
**11**	18.06	Aldehydic form of decarboxymethyl elenolic acid	215.0925	215.0931	C_10_H_16_O_5_	−2.8	Olive oil byproducts
**12**	18.39	UK 2	391.1035	391.1049	C_19_H_20_O_9_	−3.9	-
**13**	18.98	UK 3	243.0874	243.0880	C_11_H_16_O_6_	2.3	-
**14**	19.66	Elenolic acid or isomer 2	241.0718	241.0726	C_11_H_14_O_6_	−3.5	Olive oil
**15**	20.13	Hydroxytyrosol acetate	195.0663	195.0666	C_10_H_12_O_4_	−1.9	Olive oil
**16**	20.70	Hydroxy oleuropein aglycon	393.1191	393.1201	C_19_H_22_O_9_	−2.6	Olive oil and olive oil byproducts
**17**	23.16	UK 4	425.1089	425.1104	C_19_H_22_O_11_	−3.5	-
**18**	25.03	(+)-Acetoxypinoresinol	415.1398	415.1413	C_22_H_24_O_8_	−3.5	Olive oil and olive oil byproducts
**19**	25.75	Hydroxy decarboxymethyl-ligstroside aglycone	319.1187	319.1200	C_17_H_20_O_6_	4.1	Olive oil
**20**	26.17	Comselogoside	535.1457	535.1494	C_25_H_28_O_13_	−6.9	Olive oil and olive oil byproducts
**21**	31.10	6-O-[(2E)-2,6-Dimethyl-8-hydroxy-2-octenoyloxy] secologanoside	557.2240	557.2242	C_26_H_38_O_13_	−0.3	Olive oil byproducts

UK, unknown.

## Data Availability

All the data generated by this research have been included in the article. For any assistance, it is possible to contact the corresponding author.

## Supplementary Materials

The following are available online: https://www.mdpi.com/article/10.3390/foods10061407/s1, Table S1. Calibration curves used in the quantification of polar compounds present in olive oil filter cake. Table S2: Compositional variations of phenolic compounds ordered by families for each compound in all extracts, expressed in ug compound/g extract (X ± SD). Table S3: Statistical data of the drying -PLE conditions for phenolic compounds.
