# Supplementary material for: Preliminary Investigation of Different Drying Systems to Preserve Hydroxytyrosol and Its Derivatives in Olive Oil Filter Cake Pressurized Liquid Extracts
Source: Foods. 2021 Jun 18;10(6):1407. doi: 10.3390/foods10061407 (PMC8234471; doi:10.3390/foods10061407)
Supplement: Supplementary file 1 [file foods-10-01407-s001.zip › foods-1216979-supplementary.pdf]

Table S1. Calibration curves used in the quantification of polar compounds present in olive oil filter cake.

Commercial standard	Calibration range (mg/L)	Calibration curve	R^2
Quinic acid	0.5–20	y = 142862x − 99193	0.9923
Hydroxytyrosol	0.5–20	y = 33646x − 14770	0.9851
Oleuropein	0.5–20	y = 272392x − 80988	0.9977
Pinoresinol	0.5–20	y = 47383x − 36828	0.9853

Table S2. Compositional variations of polyphenols ordered by families for each compound in all extracts, expressed in ug compound/g extract (X ± SD).

Proposed compound	Drying Technique		
	Vacuum-drying	Spray-drying	Freeze-drying
**PHENOLIC COMPOUNDS**			
**Total Phenolic Alcohols**	12665 ± 48	10364 ± 243	24066 ± 488
**Total Non-oxidized Phenolic Alcohols**	6407 ± 289	3705 ± 69	13025 ± 102
Hydroxytyrosol	1548 ± 116	421 ± 15	2620 ± 83
Oxidized hydroxytyrosol	6258 ± 242	6659 ± 215	11041 ± 456
Hydroxytyrosol acetate	4859 ± 179	3284 ± 54	10405 ± 146
**Total Secoiridoids**	2320 ± 82	2202 ± 22	4654 ± 14
Secoiridoid derivative	753 ± 27	631 ± 2	1012 ± 36
Hydroxy oleuropein aglycon	620 ± 28	532 ± 11	1235 ± 15
Hydroxy decarboxymethyl-ligstroside aglycone	227 ± 11	270 ± 5	770 ± 14
Oleuropein aglycone derivative	412 ± 12	435 ± 19	961 ± 29
Comselogoside	308 ± 7	334 ± 6	676 ± 57
6-O-[(2E)-2,6-Dimethyl-8-hydroxy-2-octenoyloxy] secologanoside	NQ	NQ	NQ
**Total Phenolic Alcohols + Secoiridoids**	14986 ± 130	12566 ± 265	28720 ± 492
**Total Non-oxidized Phenolic Alcohols + Secoiridoids**	8727 ± 367	5907 ± 83	17679 ± 93
**Total Lignans**	1316 ± 39	1131 ± 19	2018 ± 36
(+)-Acetoxypinoresinol	1316 ± 39	1131 ± 19	2018 ± 36
**Total Phenolic Compounds**	16301 ± 94	13697 ± 248	30738 ± 630
**PHENOLIC COMPOUNDS DERIVATIVES (non-phenolic molecules)**			
**Total oleosides, elenolic acids and derivatives**	1601 ± 61	1470 ± 36	3047 ± 11
Hydroxylated product of the dialdehydic form of decarboxymethyl-elenolic acid	NQ	NQ	NQ
Elenolic acid or isomer 1	182 ± 6	156 ± 4	324 ± 7
Elenolic acid or isomer 2	437 ± 18	495 ± 9	961 ± 10

Aldehydic form of decarboxymethyl elenolic acid	148 ± 3	113 ± 1	423 ± 6
Dialdehydic form of decarboxymethyl elenolic acid	NQ	NQ	NQ
Oleoside	833 ± 34	706 ± 27	1338 ± 24
**OTHER POLAR COMPOUNDS**			
Quinic acid	6357 ± 619	3620 ± 40	2758 ± 212
**Total other polar compounds**	6357 ± 619	3620 ± 40	2758 ± 212

NQ, not quantitated.

Table S3. Statistical data of the Drying-PLE extraction conditions for phenolic compounds.

**Drying system**	**Hydroxytyrosol**	**Hydroxytyrosol acetate**	**Oxidized hydroxytyrosol**	**Secoiridoid derivative**	**Hydroxy oleuropein aglycon**	**Hydroxy decarboxymethyl-ligstroside aglycone**	**Oleuropein aglycone derivative**	**Comselogoside**
Spray-drying/ Vacuum-drying	1	1	0	1	1	1	0	0
Freeze-drying/ Vacuum-drying	1	1	1	1	1	1	1	1
Freeze-drying/ Spray-drying	1	1	1	1	1	1	1	1

**Drying system**	**Oleoside**	**Elenolic acid or isomer 1**	**Dialdehydic form of decarboxymethyl-elenolic acid**	**Elenolic acid or isomer 2**	**Total phenolic alcohols**	**Total secoiridoids**	**Total lignans**	**Total phenolic alcohols + secoirid**
Spray-drying/ Vacuum-drying	1	1	0	1	1	0	1	1
Freeze-drying/ Vacuum-drying	1	1	1	1	1	1	1	1
Freeze-drying/ Spray-drying	1	1	1	1	1	1	1	1

**Drying system**	**Total non oxidized phenolic alcohols + secoirids**	**Oleosides + Elenolic acids derivatives**	**Total phenolic compounds**					
Spray-drying/ Vacuum-drying	1	1	1					
Freeze-drying/ Vacuum-drying	1	1	1					
Freeze-drying/ Spray-drying	1	1	1					

1 indicated that the means difference was significant at the 0.05 level.
0 indicated that the means difference was not significant at the 0.05 level.
